# Development of a Full-Length Infectious Cdna Clone of the Grapevine Berry Inner Necrosis Virus

**DOI:** 10.3390/plants9101340

**Published:** 2020-10-11

**Authors:** Xudong Fan, Zunping Zhang, Fang Ren, Guojun Hu, Chen Li, Baodong Zhang, Yafeng Dong

**Affiliations:** National Center for Eliminating Viruses from Deciduous Fruit Trees, Research Institute of Pomology, Chinese Academy of Agriculture Sciences, Xingcheng 125100, China; fanxudong@caas.cn (X.F.); zhangzunping@caas.cn (Z.Z.); renfang@caas.cn (F.R.); huguojun@caas.cn (G.H.); caaslc@163.com (C.L.); mayday0318143@163.com (B.Z.)

**Keywords:** grapevine, virus, GINV, infectious cDNA clone

## Abstract

Grapevine berry inner necrosis virus (GINV) belongs to the genus Trichovirus in the family Betaflexiviridae. The GINV isolate LN_BETA_RS was obtained from a “Beta” grapevine (*Vitis riparia × Vitis labrusca*) exhibiting chlorotic mottling and ring spot in Xingcheng, Liaoning Province, China. To verify the correlation between GINV and grapevine chlorotic mottling and ring spot disease, we constructed an infectious cDNA clone of GINV isolate LN_BETA_RS using the seamless assembly approach. Applied treatments of agroinfiltration infectious cDNA confirmed systemic GINV infection of the *Nicotiana* *occidentalis* 37B by reverse transcription polymerase chain reaction (RT-PCR) and transmission electron microscopy, exhibiting chlorotic mottling symptoms on leaves. Infectious cDNA was also transmitted to new healthy *N. occidentalis* plants through rub-inoculation. Moreover, the cDNA clone was agroinfiltrated into “Beta” and “Thompson Seedless” grapevine plantlets, and the inoculated grapevines exhibited leaf chlorotic mottling and ringspot during the two years of observation. GINV-inoculated “Beta” grapevines had serious leaf chlorotic mottling and ringspot symptoms on the whole plant, while relatively few symptoms were observed on the leaves of agroinoculated “Thompson Seedless” grapevines in early spring and only weak ring spot gradually appeared later in the top young leaves. Our experiments fulfilled Koch’s postulates and revealed the causative role of GINV in grapevine chlorotic mottling and ring spot disease.

## 1. Introduction

China has one of the world’s leading grape-production areas, which was recently estimated to cover 725,100 ha. Grapes are widely favored by consumers and fruit growers because they have high nutritional and economic values. Grapevines can be infected by close to 80 viral species, some of which affect vine health and cause serious economic losses [1,2]. To date, 20 species of grapevine viruses have been reported in China, including grapevine leafroll-associated virus 1–4, 7 and 13, grapevine rupestris stem pitting-associated virus, grapevine fleck virus, grapevine fanleaf virus, grapevine virus A, grapevine virus B, grapevine virus E, grapevine virus T, grapevine Pinot gris virus (GPGV), grapevine berry inner necrosis virus (GINV), grapevine fabavirus (GFabV), grapevine rupestris vein feathering virus, grapevine geminivirus A, grapevine Syrah virus-1. and grapevine red globe virus [3,4,5,6,7,8,9,10,11,12,13,14,15,16,17,18,19,20,21]. Although the effects of viral diseases on grapevines have been well documented [1,22], it is still difficult to judge the pathogenicity of a single virus to a grapevine because grapevines in the vineyard are subjected to multiple simultaneous viral infections. Nevertheless, our previous studies, based on symptom investigations and virus identification in a large number of grapevine samples, showed that GINV, GPGV, and GFabV were associated with grapevine leaf mottling or leaf deformation in some grapevine cultivars and rootstocks in China [15,16,17,23,24,25].

In 1997, GINV was first identified in Japan as the possible pathogen responsible for grapevine berry inner necrosis disease [26], which had first been identified in Japan in 1984, and it was originally named as grapevine mosaic disease, one of the most economically important viral diseases of grapevine in Yamanashi, Japan [27,28]. Grapevines of susceptible cultivars grow less vigorously and sprout late in spring, and the vines show inner necrosis of shoots, shortened internodes, and various patterns of yellow leaf discoloration [26]. Berries of the diseased grapevines remain small and show discolored rinds and necrotic flesh. In the field, GINV is transmitted by grapevine erineum mites [28]. Since it was reported in Japan, GINV has only been found in China [15]. The virus is widely present in major grapevine rootstocks and cultivars in China, and it can be associated with chlorotic mottling and ring spot in “Beta” grapevines and other cultivars [15,23]. However, this association did not demonstrate disease causality because these studies have not yet satisfied all Koch’s postulates.

Phylogenetically, GINV is a member of the genus *Trichovirus* in the family Betaflexiviridae [26]. The genome of GINV contains three open reading frames and two untranslated regions at the 5′ and 3′ termini. The proteins encoded by the three open reading frames of GINV are RNA polymerase, movement protein (MP), and capsid protein (CP). Variants derived from different GINV isolates can be divided into three phylogenetic groups, and the complete genomes of three different GINV variant types have been obtained in previous studies [15,23,29]. Compared with GPGV, a close relative, GINV shows overall genetic diversity and is likely evolving under balancing selection [30].

In the present study, we constructed an infectious GINV cDNA clone on the basis of the available genome sequence of the GINV isolate LN_BETA_RS. The GINV full-length clone successfully inoculated *Nicotiana occidentalis* (37B) and grapevines, which satisfied Koch’s postulates and further confirmed the causative relationship between GINV and grapevine chlorotic mottling and ring spot disease. This study will provide the basis for the prevention and treatment of the disease of chlorotic mottling and ring spot in main grapevine rootstocks and cultivars in China, and this infectious GINV cDNA clone will also aid in further research on GINV’s pathogenicity.

## 2. Results

### 2.1. Full-Length cDNA Clones of Grapevine Berry Inner Necrosis Virus (GINV)

**Figure 1:**
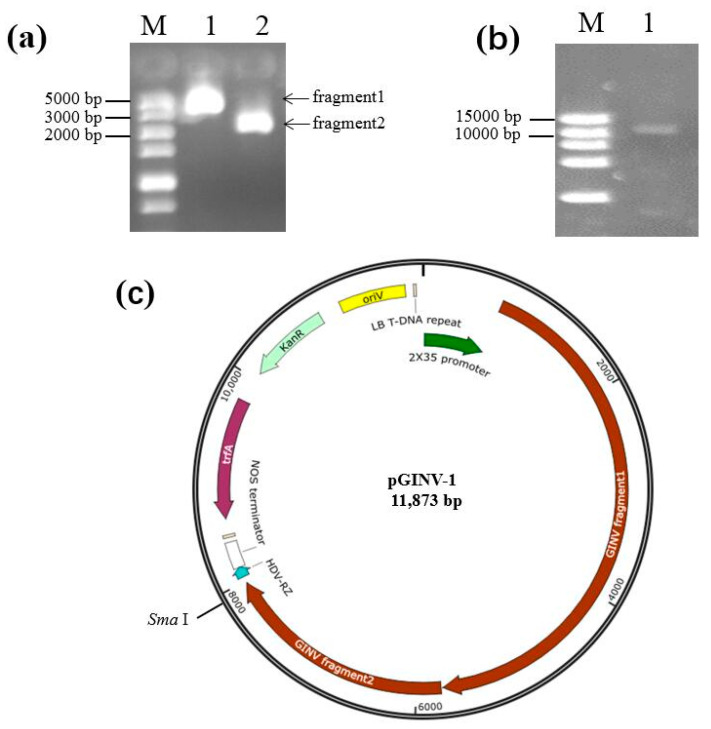
Construction of full-length cDNA clones of grapevine berry inner necrosis virus (GINV). (**a**) Two overlapping fragments covering the complete genome of grapevine berry inner necrosis virus(GINV) isolate LN_Beta_RS amplified by reverse transcription polymerase chain reaction (RT-PCR). M, DL5000 (TaKaRa, Dalian, China); (**b**) *Sma*I digested recombinant pGINV-1. M, DL15000 (TaKaRa, Dalian, China); (**c**) pGINV-1 map. oriV, origin of vegetative replication; KanR, resistance gene to kanamycin; trfA, transacting replication protein that binds to and activates oriV; NOS terminatior, nopaline synthase terminator; HDV-RZ, ribozyme of hepatitis helta virus.

Two fragments covering the complete genome of GINV isolate LN_Beta_RS were obtained by RT-PCR (Figure 1a), and they were fused through overlapping sequences, into digested pXT1 using the Seamless Assembly and Cloning kit (Aidlab, Beijing, China). The fused fragments were transformed into competent *Escherichia coli* DH5a cells, and single clones were grown on the Luria Broth (LB) liquid medium supplemented with 50 mg/L kanamycin. Positive clones were selected using PCR with two sets of primers, GINVCP1A/1B and GINVMP1A/1B, amplifying the CP and MP, respectively, and were confirmed by *Sma*I digestion of the recombinant plasmid (Figure 1b). Positive clones were sequenced to confirm the GINV sequence, and the GINV genome of the recombinant plasmid was intact, without a deletion or insertion (Figure 1c). The recombinant cloned plasmid pGINV-1 was also transformed into *Agrobacterium tumefaciens* EHA105.

### 2.2. GINV Infectious cDNA Clone Agroinoculated into N. Occidentalis 37B

**Figure 2:**
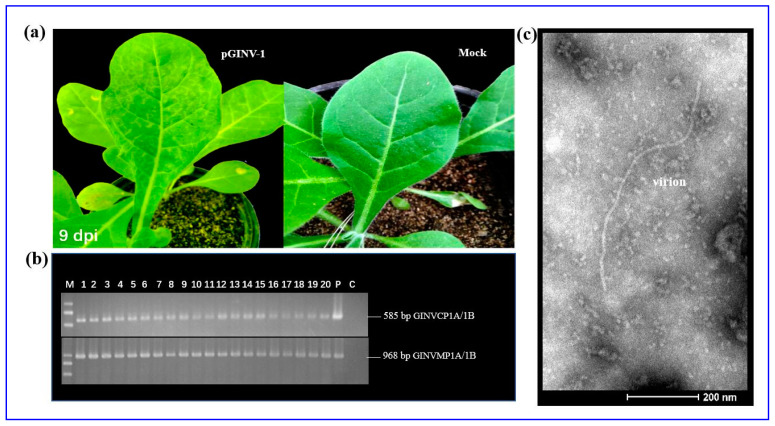
Inoculation of *N. occidentalis* plants using the GINV full-length infectious cDNA clone pGINV-1. (**a**) Infectivity test of pGINV-1 in *N.occidentalis* plants. The agroinoculated *N. occidentalis* plants behaved chlorotic mottle at 9 days postinoculation (dpi). Mock, infiltrated with agrobacterial cells containing the empty pXT1 vector; (**b**) RT-PCR detection of GINV in inoculated *N. occidentalis* plants using primer pairs GINVCP1A/1B and GINVMP1A/1B. Lanes 1–20, agroinoculated *N. occidentalis* plants. P, positive control and C, negative control; (**c**) Transmission electron micrograph of GINV in the inoculated *N. occidentalis* plants. In total, 8 intact virions were measured, and a representative micrograph is shown.

The full-length cDNA clone pGINV-1 was agroinoculated into healthy *N. occidentalis* 37B, and all 20 inoculated *N. occidentalis* plants showed obvious chlorotic mottle on two newly expanding leaves, 7–9 days after inoculation (Figure 2a). However, the symptoms became weak or disappeared on additional later expanding leaves. No symptoms developed on the inoculated leaves. Healthy control plants agroinoculated with the empty plasmid remained symptomless. Symptomatic plants tested GINV positive and control plants tested negative as assessed by RT-PCR using two sets of primers, GINVCP1A/1B and GINVMP1A/1B (Figure 2b). The virus was transmitted by rub-inoculation from the agroinoculated *N. occidentalis* 37B plants to healthy plants, resulting in the same symptoms (Figure S1). Filamentous viral particles were observed in the agroinoculated *N. occidentalis* 37B plants using transmission electron microscopy (Figure 2c).

### 2.3. GINV cDNA Clone Was Highly Infectious to Grapevines

**Figure 3:**
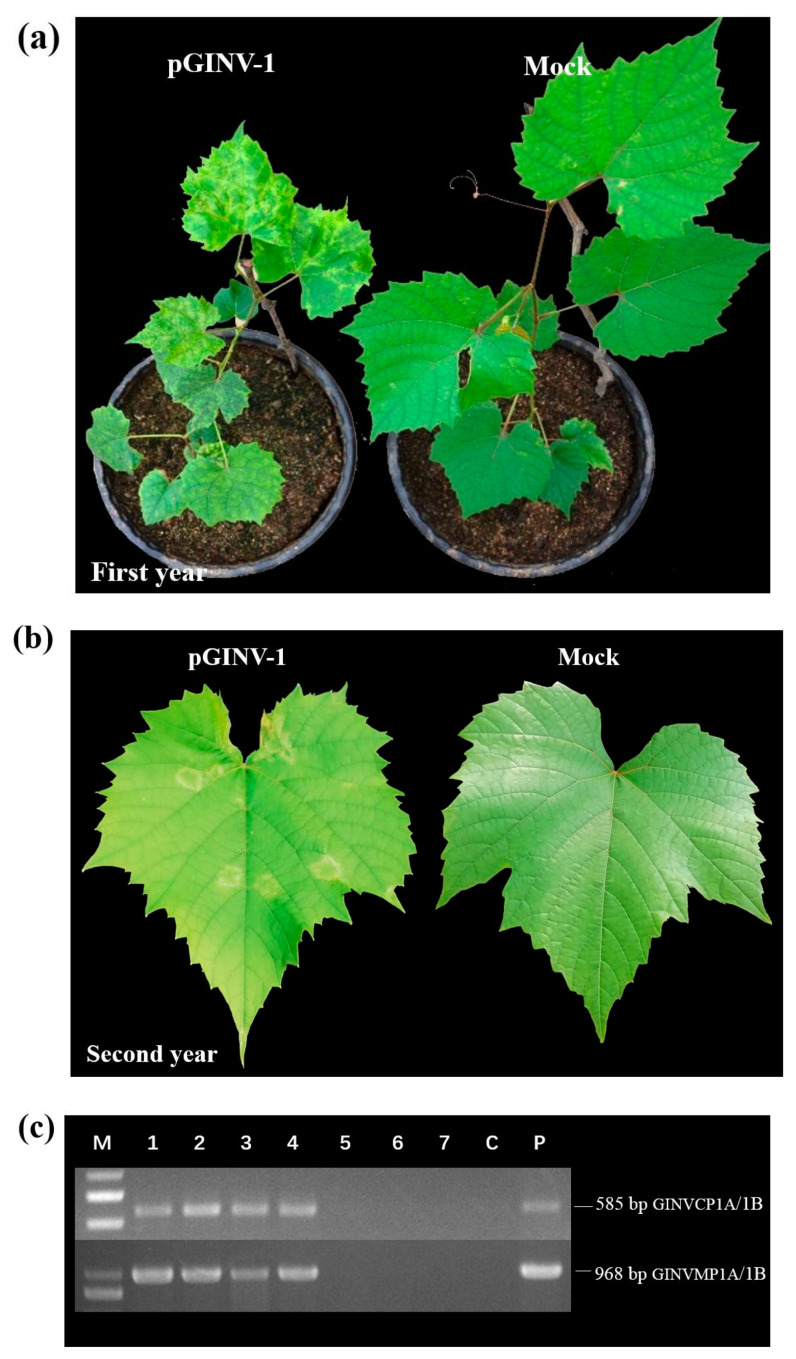
Infectivity test of pGINV-1 in “Beta” grapevines. (**a**) Agroinoculated “Beta” and control grapevines three months after agroinoculation. Mock, infiltrated with agrobacterial cells containing the empty pXT1 vector; (**b**) Agroinoculated “Beta” and control grapevines two years after agroinoculation; (**c**) RT-PCR detection of GINV in inoculated “Beta” grapevines using two GINV primer pairs. M, DL2000 (TaKaRa, Dalian, China), P, positive control and C, negative control;

**Figure 4:**
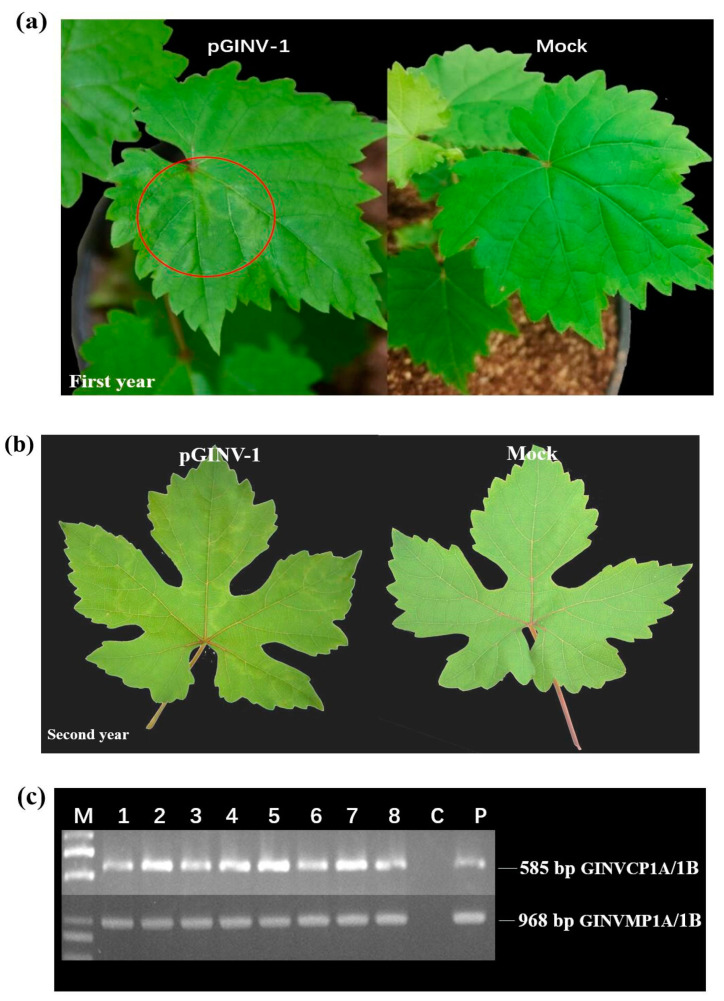
Infectivity test of pGIN1 in “Thompson Seedless” grapevines. (**a**) Agroinoculated “Thompson Seedless” and control grapevines three months after agroinoculation, Mock, infiltrated with agrobacterial cells containing the empty pXT1 vector; (**b**) Agroinoculated “Thompson Seedless” and control grapevines two years after agroinoculation; (**c**) RT-PCR detection of GINV in inoculated “Thompson Seedless” grapevines using two GINV primer pairs. M, DL2000 (TaKaRa, Dalian, China), P, positive control and C, negative control;

The infectious cDNA clone of pGINV-1 was agroinoculated independently into 10 “Beta” and 10 “Thompson Seedless” grapevine plantlets. After soaking in the agrobacterium-containing medium, the plantlets that survived were moved to a greenhouse. After three months, the newly expanding leaves of the inoculated plantlets were used for PCR-based GINV detection with primer pairs GINVCP1A/1B and GINVMP1A/1B. In total, four out of seven “Beta” plantlets tested positive for GINV as assessed by the two pairs of primers (Figure 3c), and 100% (8/8) of “Thompson Seedless” grapevines tested positive (Figure 4c), suggesting that the infectious full-length cDNA of pGINV-1 successfully infected the grapevine plantlets. The GINV-infected “Beta” grapevines showed dwarfing, systemic chlorotic mottling, and ring spot symptoms, while the control grapevines remained asymptomatic (Figure 3a). The GINV-infected “Thompson Seedless” grapevines only showed weak ring spot symptoms on young leaves (Figure 4a). In the early spring of the second year, the fresh leaves of “Beta” grapevines showed systemic chlorotic mottling, but the “Thompson Seedless’ grapevines showed no symptoms. Later, the infected “Beta” grapevines also showed ring spot on some top leaves, while the control grapevines remained asymptomatic (Figure 3b). The infected “Thompson Seedless” grapevines also showed ring spot, while the corresponding control grapevines remained asymptomatic (Figure 4b). In summer, the newly expanded leaves of both cultivars were asymptomatic.

The amplicons of GINV MP and CP genes from agroinoculated “Beta” and “Thompson Seedless” grapevine were sequenced and were compared to that of the GINV isolate LN_BETA_RS (GenBank accession no. KU234316). The sequencing result showed that the sequence of the GINV genes in the three tested agroinfected vines of every cultivar was identical to that in the isolate LN_BETA_RS.

## 3. Discussion

The construction and agroinoculation of full-length infectious cDNA clones have been used to satisfy Koch’s postulates for many plant viruses [31,32,33]. However, among viruses of woody perennial plants, only grapevine red blotch virus has been proven to be the causative infectious agent, fulfilling Koch’s postulates, using agroinoculation. It was recently confirmed as the causative pathogen of red blotch disease [34]. In our previous studies, GINV was associated with the occurrence of chlorotic mottling and ring spot symptoms in most diseased grapevine cultivars in China [23], which satisfied the first of Koch’s postulates, in which the microorganism must be present in most, if not all, cases of the disease. The second postulate states that the microorganism must be isolated from the diseased host and grown in a pure culture. For this purpose, we developed an infectious GINV clone to circumvent the inability to maintain plant viruses in pure cultures. We agroinoculated the infectious clone into vines, and “Beta” and “Thompson Seedless” grapevines showed obvious chlorotic mottling and ring spot symptoms, reproducing the original disease present in the field [15], thereby satisfying the third postulate. The fourth premise now considered as one of Koch’s postulates [35] states that the microorganism must be re-isolated from the newly diseased host and shown to be the same as the original inoculated microorganism. The sequence alignment of the GINV genes in agroinfected vines and the original GINV isolate LN_Beta_RS met this requirement. In conclusion, the present study showed that GINV is the agent causing chlorotic mottling and ring spot symptoms in “Beta” and “Thompson Seedless” grapevines, and the results fulfilled Koch’s postulates.

There are potential reasons for the limited correlation between symptomatology and viral isolates. For instance, the presence of different viral variants in the plant, interference from other viruses or pathogens in the same plant, different viral concentrations, the tolerance or resistance of different host cultivars. and external factors acting on the plant’s defenses [36]. To avoid the influences of such factors, this study established a reliable system for the study of GINV pathogenicity in controlled conditions using the GINV cDNA clone. When inoculated into grapevines, GINV caused chlorotic mottling and ring spot on the leaves of “Beta” and “Thompson Seedless” grapevines, consistent with symptoms observed in infected field-grown grapevines [15]. However, the symptoms of GINV infections differed on the two cultivars. GINV-inoculated “Beta” grapevines had serious leaf chlorotic mottling and ringspot symptoms on the whole plant, while relatively few symptoms were observed on the leaves of agroinoculated “Thompson Seedless” grapevines in early spring and only weak ring spot gradually appeared later in the top young leaves. Thus, the present study indicated that different tolerance or resistance levels of different grapevine cultivars may contribute to the varied symptoms produced when infected by the same viral variants of GINV. The use of the GINV cDNA clone may better reveal such differences as compared with viral isolates, in more grapevine cultivars. Because some latent symptoms caused by GINV can be observed in the variety “Thompson Seedless”, it is important to detect GINV in nursery production and sanitary status inspection of planting material regardless symptom visibility. The GINV cDNA clone will also be used to study interactions between GINV and different grapevine cultivars.

The GPGV cDNA clone infects *Nicotiana benthamiana* and causes leaf mottling and chlorosis [37]. In this study, the GINV cDNA clone was used to successfully infect *N. occidentalis* plants and caused chlorotic mottle, but it failed to infect *N. benthamiana*. This further indicated that two similar viruses may have different host ranges, which may be related to GPGV being widely distributed throughout the world and GINV being limited to Asia (Japan and China) [30]. Similar to GPGV-infected grapevines, symptom recovery has also been observed in GINV-inoculated *N. occidentalis* and grapevines. While the two newly unfolded leaves were symptomatic, later leaves of *N. occidentalis* were generally asymptomatic, independent of whether the plant was sap-inoculated or agroinoculated. As in a previous study [37], the growth of *N. occidentalis* in a greenhouse at 25 °C indicated that the disappearance of symptoms cannot be explained by hot temperatures, which has been reported to occur for other viral diseases [38]. Symptom recovery in GINV-infected grapevines in the field usually occurred in summer when temperatures were elevated. The present study suggested that different types of symptom recovery occur in different GINV-infected plants. Further investigations into the symptom-recovery responses of different plants or cultivars will increase our understanding of recovery induction in GINV-infected grapevines.

## 4. Materials and Methods

### 4.1. Plant Materials 

*Vitis vinifera* cultivars “Beta” and “Thompson Seedless” were selected for this study. In vitro cultured grapevine plantlets were maintained as mother materials at the National Center for Eliminating Viruses from Deciduous Fruit Tree, Research Institute of Pomology, Chinese Academy of Agriculture Sciences. The mother plants were generally tested for viruses every two years and have tested negative for GINV and other viruses reported in China three times, including grapevine leafroll-associated virus 1–4, 7, and 13, grapevine rupestris stem pitting-associated virus, grapevine fleck virus, grapevine fanleaf virus, grapevine virus A, grapevine virus B, grapevine virus E, GPGV, GFabV, grapevine red blotch virus, grapevine rupestris vein feathering virus, grapevine geminivirus A, grapevine Syrah virus-1, and grapevine red globe virus. To exclude other recently reported viruses, the mother plantlets were also subjected to small RNA sequencing and no viruses were found in both cultivars. The grapevine plantlets bred from the mother plants never showed any symptoms of viral disease after being transferred to the field through visual observation.

### 4.2. Viral Isolate Sources

The previously reported isolate LN_Beta_RS (GenBank accession no. KU234316) was used as the template to construct the infectious full-length cDNA clones of GINV. The GINV isolate LN_Beta_RS is closely associated with chlorotic mottling and ring spot symptoms of “Beta” and ’Thompson Seedless’ grapevines [15].

### 4.3. Construction of Full-Length cDNA Clones

The binary vector pCB301-2×35S-MCS-HDVRZ-NOS, plasmid pXT1 (GenBank accession no. JN029690) [39], was used in this study, and it possesses two CaMV 35S promoters and the hepatitis delta viral ribozyme sequence followed by a CaMV 35S polyadenylation signal. The vector was linearized by double digestion with *Stu*I and *Sma*I restriction enzymes.

Two overlapping DNA fragments covering the complete genome of GINV isolate LN_Beta_RS were prepared by RT-PCR. The total RNA obtained for small RNA sequencing in a previous study [40] was used for the amplification of GINV isolate LN_Beta_RS. The extracted RNA was reverse transcribed using Superscript II (Invitrogen, Shanghai, China) with Oligo(dT)_18_, for 1 h 30 min, at 42 °C. A cDNA fragment of 4998 bp, corresponding to the 5′ part of the viral genome, was amplified using the primer pair Fragment1F: CATTTCATTTGGAGAGGCCTGATAAGTAGTTGACAGTGATCAATTG/Fragment1R: AGACATCAAAGGCCTCGTAATCAGATTC. A cDNA fragment of 2325 bp, corresponding to the 3′ part of the viral genome, was amplified using the primer pair Fragment2F: CGAGGCCTTTGATGTCTCACAGGACC/Fragment2R: ATGCCATGCCGACCCGGGT _(40)_GGAAATTAATTAAAAC. The cDNA fragments were generated using the Phusion^®^ High-Fidelity PCR Kit (cat. no. E0553S, NEB, Beijing, China), following the manufacturer’s instructions.

The digested PCB301 vector and the two overlapping DNA fragments of GINV were purified using a Gel Extration kit (Aidlab, Beijing, China). The gel-purified fragments were fused using a Seamless Assembly and Cloning kit (Aidlab, Beijing, China). After the reaction, 5 µL of the mixture was used directly in the transformation of competent *Escherichia coli* DH5a cells (TaKaRa, Dalian, China), and then they were transferred to LB solid medium supplemented with 50 mg//L kanamycin.

Positive clones were selected on LB liquid medium supplemented with 50 mg/L kanamycin. Two primer pairs, GINVCP1A/1B and GINVMP1A/1B [23], amplifying the CP and MP, respectively, were used for the screening of colonies. The plasmids in the positive colonies were extracted using a HighPure Plasmid Mini Kit (Aidlab, Beijing, China) and confirmed by *Sma*I digestion. To guarantee the correctness of the GINV genome sequence, three clones were also sequenced. After confirmation, the plasmids were delivered into competent *A. tumefaciens* strain EHA105 using the freeze–thawing method.

### 4.4. Agroinoculation of Herbaceous Plants

*Agrobacterium tumefaciens* strain EHA105 was incubated in LB medium containing 25 mg/L rifampicin and 50 mg/L kanamycin at 28 °C, with shaking until reaching an OD_600_ of 1. Agrobacterial cells were pelleted at 8000 rpm, resuspended in infiltration buffer containing 100 µM acetosyringone, 10 mM MgCl_2_, and 10 mM MES solution and adjusted to an OD_600_ of 1. After induction for 3.5 h, agrobacteria were injected into two leaves of four-leaf-stage *N. occidentalis* 37B using 1 mL syringes without needles. Plants infiltrated with *Agrobacterium* containing empty pXT1 served as the mock control. The symptom development was observed for three months after inoculation. The presence of GINV in these inoculated plants was confirmed by RT-PCR and electron microscopy.

### 4.5. Agroinoculation of Grapevine Plantlets

The procedures for inoculating grapevine plantlets were carried out in accordance with previously reported methods [40]. *Agrobacterium tumefaciens* strain EHA105 containing the full-length cDNA clone was incubated to an OD_600_ of 1. Agrobacterial cells were pelleted and resuspended in half-strength Murashige and Skoog (MS) liquid medium supplemented with 1 mg/L 6-benzylaminopurine, 0.5 mg/L α-naphthyacetic acid, and 100 M acetosyringone to an OD_600_ of 1. “Beta” and “Thompson Seedless” grapevine plantlets, growing in vitro and having three to five fresh roots each, were detached from the solid medium. Then, the roots were gently injured using a sterile needle. Next, the plantlets were transferred to the centers of sterile Whatman No. 1 filter papers (Whatman, Beijing, China) and immersed in 20 mL half-strength MS liquid medium containing *A. tumefaciens*. The plantlets were maintained in the liquid media for 10 days, and then transferred to fresh MS liquid medium for another 14 days. Finally, the plantlets were transferred to fresh solid medium supplemented with 500 mg/L cefotaxime and maintained for subsequent analyses.

### 4.6. RT-PCR and Sequencing

In inoculated plants, the presence of GINV was assessed by RT-PCR. Total RNAs of the tested plants were extracted using a previously reported method [41]. RT-PCR was used to detect GINV using primer pairs GINVCP1A/1B and GINVMP1A/1B, amplifying the CP and MP, respectively, in accordance with a previously published method [23]. The amplicons were cloned individually into the pTOPO-TA clone vector (Aidlab, Beijing, China). At least three clones were sequenced by the Sangon Biotech company (Shanghai, China).

### 4.7. Electron Microscopy 

Particles of GINV were observed in the tested plants using a negative staining method. A few leaves from the inoculated plants were placed on clean slides, 2.5% glutaraldehyde solution was added, and then the leaves were quickly cut using a blade. A grid was held in forceps and its surface placed close to the sap for 3 min. Then, the sap was drawn from the edge of the grid using filter paper. The grid was stained with 1% phosphotungstic acid (pH 6.5) for 3 min and excess stain was drawn from the edge of the grid using filter paper. Afterward, the grid was placed directly into a grid box and allowed to air dry at room temperature. The dry grid was observed using a transmission electron microscope (Tecnai G2 Spirit, FEI, Hillsboro, OR, USA).

## Supplementary Materials

The following are available online at https://www.mdpi.com/2223-7747/9/10/1340/s1, Figure S1: Rub-inoculation of healthy *N. occidentalis* 37B plants using the sap of agroinoculated *N. occidentalis* 37B plant.
