# Supplementary material for: Development of a Full-Length Infectious Cdna Clone of the Grapevine Berry Inner Necrosis Virus
Source: Plants (Basel). 2020 Oct 11;9(10):1340. doi: 10.3390/plants9101340 (PMC7601338; doi:10.3390/plants9101340)
Supplement: Supplementary file 1 [file plants-09-01340-s001.pdf]

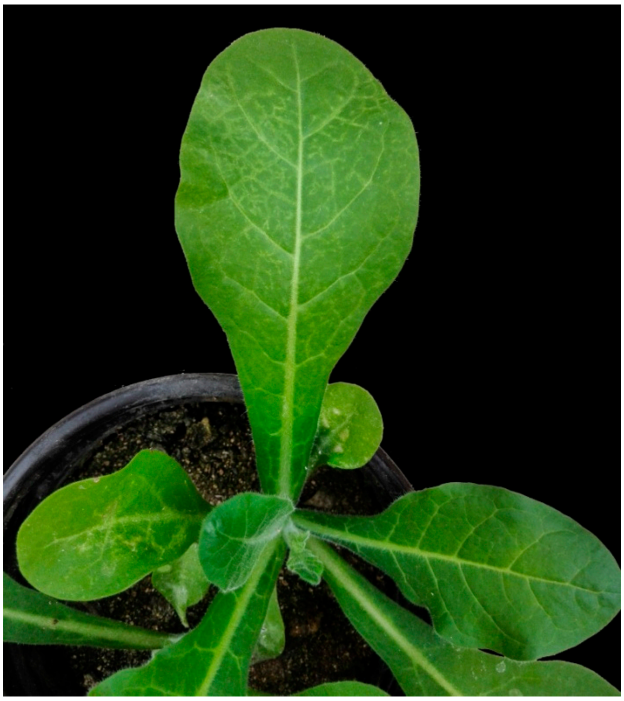

Figure S1. Rub-inoculation of healthy *N. occidentalis* 37B plants using the sap of agroinoculated *N. occidentalis* 37B plant.
